# The mediating role of sleep in the fish consumption – cognitive functioning relationship: a cohort study

**DOI:** 10.1038/s41598-017-17520-w

**Published:** 2017-12-21

**Authors:** Jianghong Liu, Ying Cui, Linda Li, Lezhou Wu, Alexandra Hanlon, Jennifer Pinto-Martin, Adrian Raine, Joseph R. Hibbeln

**Affiliations:** 1 University of Pennsylvania Schools of Nursing and Medicine, 418 Curie Blvd., Claire M. Fagin Hall, Room 426, Philadelphia, PA 19104 USA; 2 Brigham and Women’s Hospital, 75 Francis Street, Boston, MA 02115 USA; 3 University of Pennsylvania School of Arts and Sciences, 483 McNeil Building 3718 Locust Walk, Philadelphia, PA USA; 4 Acting Chief, Section on Nutritional Neurosciences LMBB, NIAAA, NIH, 5625 Fishers Lane, Rm 3N-07, MSC 9410, Bethesda, MD 20892 USA

## Abstract

Greater fish consumption is associated with improved cognition among children, but the mediating pathways have not been well delineated. Improved sleep could be a candidate mediator of the fish-cognition relationship. This study assesses whether 1) more frequent fish consumption is associated with less sleep disturbances and higher IQ scores in schoolchildren, 2) such relationships are not accounted for by social and economic confounds, and 3) sleep quality mediates the fish-IQ relationship. In this cohort study of 541 Chinese schoolchildren, fish consumption and sleep quality were assessed at age 9–11 years, while IQ was assessed at age 12. Frequent fish consumption was related to both fewer sleep problems and higher IQ scores. A dose-response relationship indicated higher IQ scores in children who always (4.80 points) or sometimes (3.31 points) consumed fish, compared to those who rarely ate fish (all *p* < 0.05). Sleep quality partially mediated the relationship between fish consumption and verbal, but not performance, IQ. Findings were robust after controlling for multiple sociodemographic covariates. To our knowledge, this is the first study to indicate that frequent fish consumption may help reduce sleep problems (better sleep quality), which may in turn benefit long-term cognitive functioning in children.

## Introduction

The long-chain omega-3 fatty acids eicosapentaenoic acid (EPA) and docosahexaenoic acid (DHA) are essential nutrients primarily found in fish^1^ and have gained increasing attention for potential health benefits ranging from cardiovascular to mental health^2,3^. As omega-3 fatty acids are known to play critical roles in the growth and functioning of neural tissue^1^, their effects on cognitive outcomes are of particular interest. Maternal fish intake or fish oil supplementation during pregnancy, for instance, is associated with improved neurodevelopmental outcomes in infants and young children, including language and visual motor skills at 6 and 18 months^4^, eye and hand coordination at age 2.5 years^5^, and IQ at age 4 years^6^. Dietary fish and omega-3 fatty acid intake is also associated with improved cognitive and academic performance in adolescents^7–9^ and reduced cognitive decline and dementia in older age^10–12^.

While animal models have demonstrated the role of omega-3 fatty acids on cognitive processes on a more molecular level^13,14^, our knowledge regarding how they improve observed cognitive performance remains limited. One pathway that has yet to be explored is sleep. Sleep is well studied in its association with cognitive function in both children^15–17^ and adults^18,19^, with insufficient or poor quality sleep being associated with poor school performance and objective measures of learning and memory^15,20^. Sleep itself is also affected by omega-3 fatty acids via several mechanisms. Animal studies have suggested the potential role of DHA in regulating endogenous melatonin production^21–23^ which has been shown to regulate circadian rhythm and improve sleep organization^24^ as well as CNS maturity in infants^25,26^. Additionally, essential fatty acids have been involved with the production of prostaglandins. Prostaglandins are believed to be the most potent endogenous sleep-promotion substance and are well known to mediate sleep/wake regulation^27^ and responses of synaptic circuitry to sleep deprivation^28^. Epidemiological studies have also demonstrated significant associations between increased fish intake and improved sleep measures in adults^29,30^ as well as infants^25,26^ and children^31^.

In light of the relationship between sleep and cognition, as well as the growing recognition that omega-3 fatty acids may lead to both improved sleep quality and cognitive outcomes, the possibility that sleep acts as a potential mediator between fish intake and improved cognition warrants further exploration and consideration. However, to our knowledge, no study has simultaneously examined how dietary fish and omega-3 fatty acid intake affects sleep and cognition. Furthermore, studies of dietary omega-3 fatty acid consumption in school-aged children examining cognition^4,7–9^ and sleep^31^ have primarily been limited to Western countries, with the latter relationship only reported by one study to date in healthy school-aged children^31^.

The present study aims to address these gaps and add to the current literature by examining dietary fish intake, sleep quality, and cognitive outcomes in a large sample of healthy, Chinese schoolchildren. The purpose of this study is thus to examine the following hypotheses: 1) frequent fish intake is linked to better sleep and long-term cognitive outcomes; 2) such relationships are robust to sociodemographic covariates; and 3) sleep mediates the fish intake and long-term cognitive outcome relationship.

## Methods

### Study population

This longitudinal study consisted of a sample of 541 Chinese school children (54% boys and 46% girls) aged 12 years from the second wave of the China Jintan Cohort Study, an ongoing prospective longitudinal study. Details on sampling at baseline and research procedures have been published elsewhere^32,33^. Of 1009 children who were followed up in the second wave (2011–2013), 541 participants who completed a self-reported food frequency questionnaire, IQ measurement, and sleep quality evaluation were included in the present study. With the exception of father’s education and home location, there were no significant differences in social demographic features between children with and without complete data. Written informed consent was obtained from parents, and approval from Institutional Review Boards was obtained from the University of Pennsylvania and the ethical committee for research at Jintan Hospital in China. All research was performed in accordance with the relevant guidelines and regulations.

### Measures

#### Fish consumption at age 9–11

A self-administrated food frequency questionnaire was used to collect information on diverse food intake, including fish consumption, when children were enrolled in 4th, 5th, and 6th grades. Fish intake frequency was measured by asking children the following question: “How often do you consume fish in a typical month? 1 = never, 2 = seldom (less than 2 times per month), 3 = sometimes (2–3 times per months), 4 = often (at least once per week)”. After preliminary analysis, categories 1 and 2 were combined due to very few “never” responses. Therefore, our analysis is based on three levels of fish consumption: “often”, “sometimes”, and “never or seldom”.

#### Sleep quality at age 9–11

Sleep quality was measured by the total sleep disturbance score, derived from parents’ report of sleep patterns in the Children’s Sleep Habits Questionnaire (CSHQ). The CSHQ consists of 33 sleep-disturbance items, which are conceptually grouped into 8 subscales: bedtime resistance, sleep-onset delay, sleep duration, sleep anxiety, night waking, parasomnias, sleep-disordered breathing, and daytime sleepiness. Parents were asked to rate each item on a 3-point scale: “usually” if the sleep pattern occurred five to seven times/week; “sometimes” two to four times/week; and “rarely” zero to one time/week in a typical week during the past month. A total sleep disturbance score was calculated as the sum of all eight subscale scores, with higher values indicating more sleep disturbance and poor sleep quality^34^. The Chinese version of the CSHQ has displayed satisfactory psychometric properties in the assessment of sleep problems in Chinese children^35^ and has been widely used^36–38^. 

#### Cognition (IQ) at age 12

IQ assessments were performed using the Chinese version of the Wechsler Intelligence Scale for Children-Revised (WISC-R). The WISC-R consists of six verbal subtests (Information, Comprehension, Arithmetic, Vocabulary, Similarities and Digit Span) that are summed to form Verbal IQ, and six non-verbal subtests (Picture Arrangement, Picture Completion, Object Assembly, Block Design, Coding and Mazes), that are summed to form Performance IQ. The Verbal and Performance IQs are combined to produce a Full-Scale IQ score. The Chinese version of WISC-R has long been standardized and shown to be reliable among Chinese children^39^. In the present study, all IQ tests were administered by two intensively-trained researchers to minimize possible investigator bias. Details of IQ test procedures have been reported elsewhere^40,41^.

#### Covariates

Sociodemographic and other relevant information collected at baseline was used as covariates in the current study; they include gender, parental education, parental occupation, parental marriage status, maternal age at childbirth, feeding type during infancy (breastfed or bottle-fed), breastfeeding duration, home location (city, town, or countryside), and siblings (yes/no). Parental education was categorized into three groups: less than high school, high school, and college or higher. Parental occupation was collapsed into unemployment, working class, and professional class. In addition, since our previous research has shown breakfast intake as an important protective predictor for cognitive function, breakfast consumption was included in the analysis as a controlled confounder.

### Statistical analysis

Baseline characteristics of child participants and their families were summarized using descriptive statistics (mean/standard deviation, median/interquartile range, and frequencies/percentages, as appropriate). Comparisons across fish consumption groups were accomplished using chi-square statistics or Fishers Exact tests and one-way ANOVA models or nonparametric Kruskal-Wallis models for categorical and continuous measures, respectively. Bivariate associations of IQ measures and total sleep disturbance scores with various baseline covariates were evaluated using general linear modeling (GLM). Robust variance estimation was used in all GLM analyses to account for possible correlations within geographic region (preschools and primary schools). GLM analyses were also applied to assess the associations of IQ measures with fish consumption frequency and total sleep disturbance score. Multivariable GLM analysis adjusted for possible confounders such as gender, father’s education, mother’s education, siblings, home location, and breakfast consumption habits. Finally, a 4-step mediation analysis was conducted to evaluate if total sleep disturbance mediates the association between fish consumption habit and IQ measures^42^. All analyses were performed using SAS 9.2^43^; two-sided *p* values less than 0.05 were considered statistically significant.

### Data availability

The datasets analyzed during the current study are available from the corresponding author upon reasonable request.

## Results

### Basic characteristics of study population

**Figure 1:**
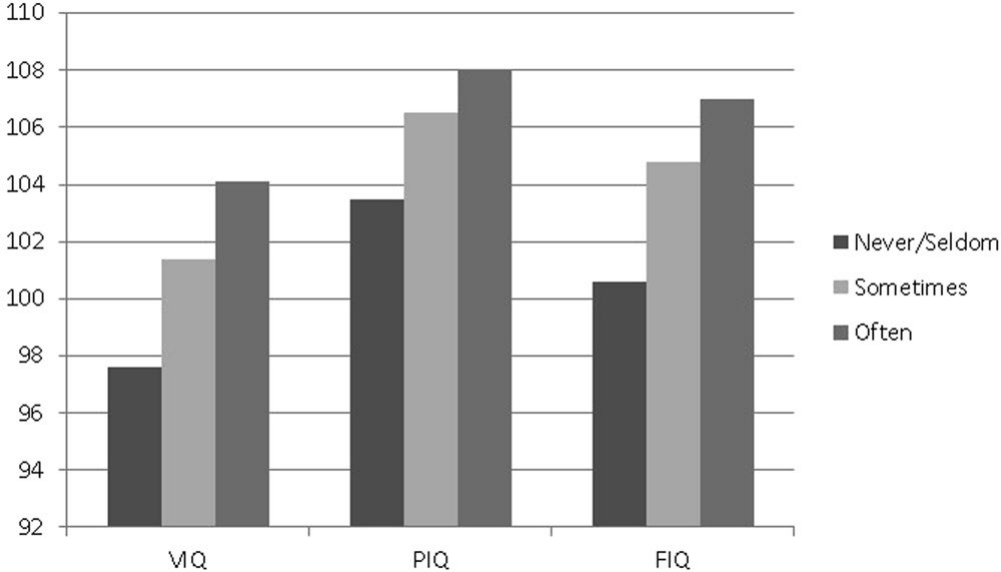
Means of IQ measures by fish consumption groups.

Of 541 schoolchildren aged 12 years, 137 (25.3%) reported consuming fish often (at least once per week), 315 (58.2%) reported eating fish sometimes (2-3 times per months), and 89 (16.5%) never or seldom ate fish (less than 2 times per month). With the exception of home location (*p* = 0.038), there were no significant differences in baseline socio-demographic characteristics by fish consumption (Table 1). IQ measures and the total sleep disturbance score demonstrated a significant association with fish consumption: as compared to children who never or seldom ate fish, those having more frequent fish intake had higher verbal, performance, and full scale IQ scores, as well as a lower total sleep disturbance score (all *p* < 0.05). Distributions of average IQ scores by fish consumption groups are displayed in Fig. 1.

**Table 1:** Baseline characteristics of school children by fish consumption habits.

	Total (n = 541)*	Fish consumption			p-value
		Never or seldom (n = 89)	Sometimes (n = 315)	Often (n = 137)	
Gender (Male)	279 (51.6)	46 (51.7)	158 (50.2)	75 (54.7)	0.669
Father’s education					0.084
Less than high school	180 (34.6)	37 (44.1)	109 (35.7)	34 (25.8)	
High school	179 (34.4)	26 (31.0)	102 (33.4)	51 (38.6)	
College or higher	162 (31.1)	21 (25.0)	94 (30.8)	47 (35.6)	
Mother’s education					0.262
Less than high school	258 (49.4)	51 (60.0)	148 (48.5)	59 (44.7)	
High school	164 (31.4)	21 (24.7)	99 (32.5)	44 (33.3)	
College or higher	100 (19.2)	13 (15.3)	58 (19.0)	29 (22.0)	
Father’s occupation					0.080
Unemployment	22 (4.4)	3 (3.6)	8 (2.8)	11 (8.4)	
Worker	275 (55.2)	51 (61.5)	158 (55.6)	66 (50.4)	
Professional	201 (40.4)	29 (34.9)	118 (41.6)	54 (41.2)	
Mother’s occupation					0.395
Unemployment	135 (26.6)	22 (26.5)	76 (25.8)	37 (28.7)	
Worker	223 (44.0)	43 (51.8)	129 (43.7)	51 (39.5)	
Professional	149 (29.4)	18 (21.7)	90 (30.5)	41 (31.8)	
Parents’ divorce/separation (No)	471 (97.7)	74 (98.7)	275 (97.2)	122 (98.4)	0.629
Maternal age at childbirth	26 (24, 27)	26 (24, 28)	26 (24, 27)	26 (25, 27)	0.604
Feed type during infancy					0.522
Breastfeeding	484 (95.1)	80 (95.2)	280 (94.3)	124 (96.9)	
Formula	25 (4.9)	4 (4.8)	17 (5.7)	4 (3.1)	
Breastfeeding duration	8.82 ± 3.04	8.99 ± 3.54	8.86 ± 2.94	8.63 ± 2.94	0.665
Home location					0.038
Countryside	62 (11.9)	7 (8.2)	43 (14.1)	12 (9.1)	
Town	87 (16.7)	20 (23.5)	39 (12.8)	28 (21.2)	
City	373 (71.5)	58 (68.2)	233 (73.1)	92 (69.7)	
Siblings					0.265
No siblings	387 (81.1)	60 (75.0)	226 (83.1)	101 (80.8)	
Have at least one sibling	90 (18.9)	20 (25.0)	46 (16.9)	24 (19.2)	
Breakfast consumption					0.308
0–2 d/w	21 (3.9)	4 (4.6)	15 (4.9)	2 (1.5)	
3–5 d/w	63 (11.8)	14 (15.9)	34 (11.0)	15 (11.0)	
6-7 d/w	450 (84.3)	70 (79.6)	260 (84.1)	120 (87.6)	
Total sleep disturbance	42.6 (38.0, 47.0)	44.7 (39.2, 51.0)	43.0 (38.0, 47.0)	42.0 (38.0, 46.0)	0.013
VIQ	101.4 ± 11.9	97.6 ± 11.7	101.4 ± 12.3	104.1 ± 10.1	<0.001
PIQ	106.4 ± 12.1	103.5 ± 12.7	106.5 ± 12.2	108.0 ± 11.3	0.024
FIQ	104.6 ± 11.9	100.6 ± 12.0	104.8 ± 12.2	107.0 ± 10.5	<0.001

Note: Proportions may not add to 100% due to rounding and sum across nominal variables may not add to 541 due to missing data. Nominal variables are shown as count (column percent); numeric variables having a skewed distribution are presented as median (inter-quartile range); normally distributed variables are shown as mean +/− standard deviation.

Fish consumption: “often” = at least once per week, “sometimes” = 2–3 times per month, “never or seldom” = less than 2 times per month. Abbreviation: VIQ, verbal IQ; PIQ, performance IQ, FIQ, full IQ.

Simple bivariate associations between total sleep disturbance and the three IQ scores with demographic and relevant covariates are summarized in Table 2. While gender, parental education and occupation, and home location were consistently associated with the three IQ measures, only the number of siblings and breakfast consumption habits were significantly associated with verbal and full scale IQ. Moreover, the total sleep disturbance score was found to be significantly associated with parental education and occupation, maternal age at childbirth, home location, and number of siblings.

**Table 2:** Bivariate general linear model (GLM) analysis: crude associations of IQ and total sleep disturbance score with demographic and relevant characteristics.

	VIQ		PIQ		FIQ		Total sleep disturbance	
	β (SE)	p-value	β (SE)	p-value	β (SE)	p-value	β (SE)	p-value
Gender (male)	1.878 (0.919)	0.041	3.926 (0.910)	<0.001	3.253 (0.913)	<0.001	−0.172 (0.830)	0.836
Father’s education								
High school	3.970 (1.072)	<0.001	2.390 (1.098)	0.030	3.890 (1.067)	<0.001	−2.014 (1.043)	0.054
College or higher	8.951 (1.105)	<0.001	5.488 (1.133)	<0.001	8.565 (1.102)	<0.001	−2.512 (1.038)	0.016
Mother’s education								
High school	5.506 (1.030)	<0.001	3.599 (1.062)	<0.001	5.511 (1.030)	<0.001	−3.041 (0.974)	0.002
College or higher	9.192 (1.193)	<0.001	4.907 (1.230)	<0.001	8.375 (1.193)	<0.001	−3.143 (1.083)	0.004
Father’s occupation								
No job	−6.611 (2.275)	0.004	−4.800 (2.255)	0.034	−6.843 (2.239)	0.002	2.935 (2.622)	0.264
Worker	−5.170 (0.981)	<0.001	−3.823 (0.972)	<0.001	−5.337 (0.966)	<0.001	2.380 (0.886)	0.007
Mother’s occupation								
No job	−5.713 (1.242)	<0.001	−4.031 (1.240)	0.001	−5.813 (1.222)	<0.001	0.926 (1.137)	0.416
Worker	−6.076 (1.117)	<0.001	−5.024 (1.116)	<0.001	−6.512 (1.101)	<0.001	2.095 (1.001)	0.037
Parent’s divorce/separation (no)	1.963 (2.824)	0.487	−1.206 (2.861)	0.673	1.161 (2.913)	0.690	0.102 (2.632)	0.969
Maternal age at childbirth	0.236 (0.174)	0.175	0.277 (0.172)	0.108	0.330 (0.173)	0.057	−0.463 (0.151)	0.002
Feed type during infancy								
Breastfeeding	3.473 (2.009)	0.084	3.152 (2.017)	0.119	3.722 (2.004)	0.064	−0.660 (1.653)	0.690
Breastfeeding duration	−0.023 (0.157)	0.884	−0.261 (0.158)	0.099	−0.139 (0.157)	0.376	0.174 (0.149)	0.243
Home location								
Countryside	−4.306 (1.438)	0.003	−4.531 (1.426)	0.002	−5.415 (1.424)	<0.001	2.153 (1.234)	0.082
Town	−3.631 (1.208)	0.003	−4.163 (1.198)	<0.001	−4.655 (1.190)	<0.001	6.285 (1.174)	<0.001
Siblings								
Have at least one sibling	−5.418 (1.217)	<0.001	−1.054 (1.211)	0.385	−3.664 (1.211)	0.003	3.360 (1.137)	0.003
Breakfast consumption								
6–7 d/w	7.752 (2.544)	0.002	0.741 (2.721)	0.785	5.438 (2.655)	0.041	−0.719 (2.489)	0.773
3–5 d/w	4.952 (2.872)	0.085	−0.540 (3.072)	0.861	2.810 (2.996)	0.349	2.348 (2.716)	0.388

Reference groups used in GLM analysis were: female (gender), less than high school (education), professional (occupation), yes (parent’s divorce or separation), formula (feed type during infancy), city (growing area), no siblings (siblings), and 0–2d/w (breakfast consumption). β denotes estimated regression coefficients.

### Fish consumption and cognitive function

Dose-response relationships between fish consumption frequency and IQ scores were observed in GLM analyses with and without adjustment for selected covariates (Table 3). Multivariable analyses indicate that, among children aged 12 years, those who frequently consumed fish when they were aged 9–11 years scored 4.75 points higher in verbal IQ (*p* = 0.002; Cohen’s d = 0.595), 3.79 points higher in performance IQ (*p* = 0.026; Cohen’s d = 0.416), and 4.80 points higher in full scale IQ (*p* = 0.003; Cohen’s d = 0.567), compared to those who never or seldom consumed fish. Similarly, children who sometimes consumed fish demonstrate verbal, performance, and full scale IQ scores 2.92 (*p* = 0.036, Cohen’s d = 0.317), 2.52 (*p* = 0.097, Cohen’s d = 0.236), and 3.31 (*p* = 0.023, Cohen’s d = 0.347) points higher than those who never or seldom consumed fish, respectively.

**Table 3:** Bivariate and multivariable general linear model (GLM) analysis: associations among fish consumption, total sleep disturbance score and IQ measures.

	VIQ		PIQ		FIQ		Total sleep disturbance	
	β (SE)	p-value	β (SE)	p-value	β (SE)	p-value	β (SE)	p-value
Bivariate models								
Fish consumption								
Often (n = 137)	6.56 (1.59)	<0.001	4.50 (1.64)	0.006	6.40 (1.61)	<0.001	−5.57 (1.32)	<0.001
Sometimes (n = 315)	3.79 (1.40)	0.007	2.92 (1.45)	0.044	4.22 (1.42)	0.003	−4.24 (1.20)	<0.001
Never or seldom (n = 89)	ref	ref	ref	ref	ref	ref	ref	ref
Total sleep disturbance	−0.26 (0.06)	<0.001	−0.21 (0.06)	0.001	−0.25 (0.06)	<0.001	—	—
Multivariable models								
Fish consumption								
Often (n = 137)	4.75 (1.55)	0.002	3.79 (1.69)	0.026	4.80 (1.63)	0.003	−4.49 (1.38)	0.001
Sometimes (n = 315)	2.92 (1.39)	0.036	2.52 (1.51)	0.097	3.31 (1.45)	0.023	−3.01 (1.28)	0.019
Never or seldom (n = 89)	ref	ref	ref	ref	ref	ref	ref	ref
Total sleep disturbance	−0.17 (0.06)	0.007	−0.16 (0.07)	0.019	−0.19 (0.067)	0.005	—	—

All multivariable models adjusted for gender, father’s education, mother’s education, siblings, home location, and breakfast consumption habits. β denotes estimated regression coefficients.

### Fish consumption and sleep quality

More frequent fish eating was found to be independently associated with less sleep disturbances, which indicated better overall sleep quality. After controlling for possible confounding, children who often consumed fish and those eating fish sometimes had a total sleep disturbance score 4.49 (*p* = 0.001; Cohen’s d = 0.221) and 3.01 (*p* = 0.019; Cohen’s d = 0.132) points lower, respectively, than those who never or seldom ate fish (Table 3).

### Sleep quality and cognitive function

Children with fewer sleep disturbance problems were more likely to have higher cognitive functioning. The negative associations between total sleep disturbance score and three IQ measures are summarized in Table 3. Multivariable GLM analysis showed that among schoolchildren aged 12 years, a 1 point decrease in the total sleep disturbance score was associated with 0.17, 0.16, and 0.19 point increases in verbal, performance, and full scale IQ scores, respectively (all *p* < 0.05).

### Sleep quality partially mediates the association between fish consumption and cognitive functioning

**Figure 2:**
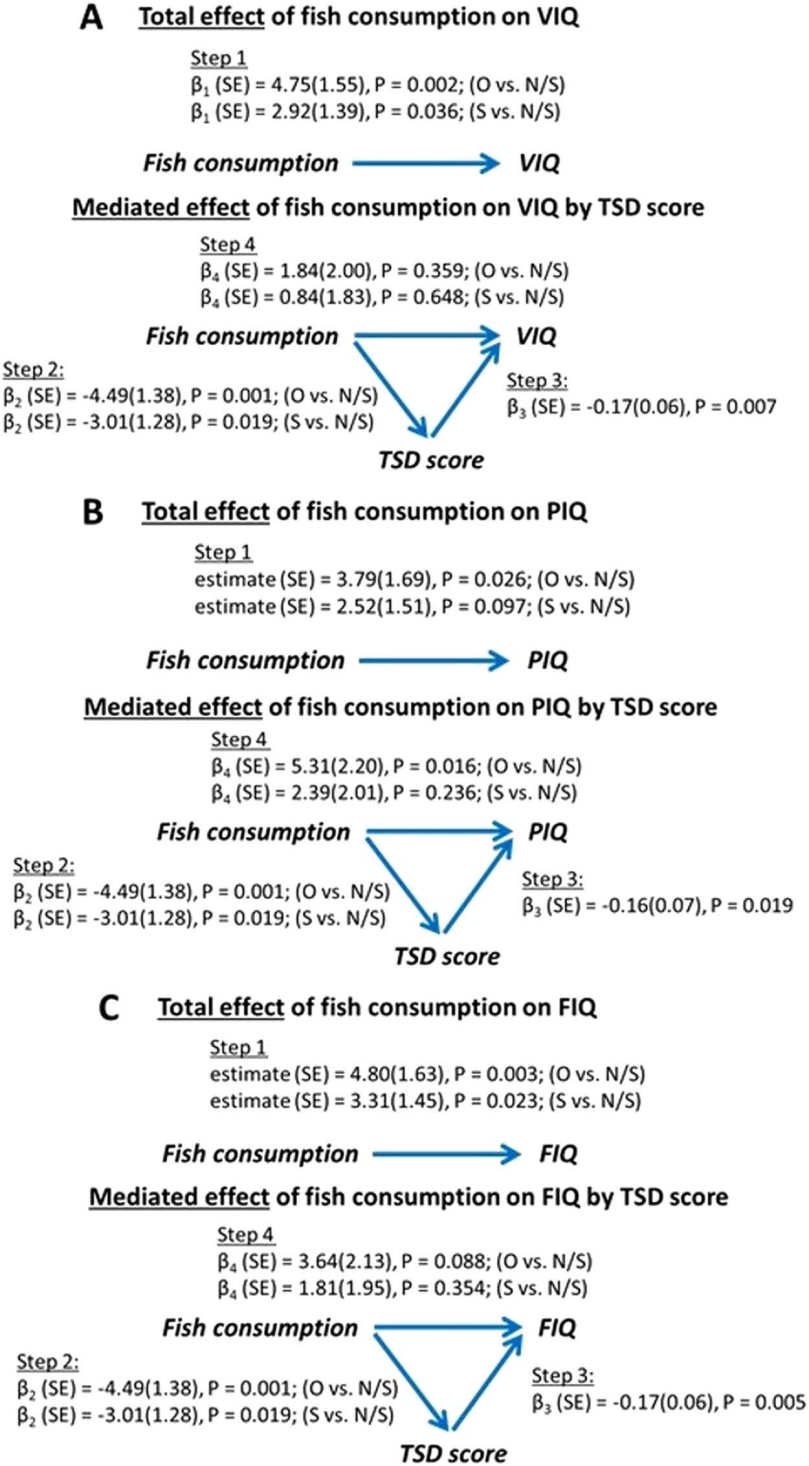
Total and mediated effect of fish consumption on IQ scores. Note: All multivariable models adjusted for gender, father’s education, mother’s education, siblings, home location, and breakfast consumption habits. Reference group: never or seldom (fish consumption). Acronym: O, often; S, sometimes; N/S, never or seldom; β, estimated regression coefficient; SE, standard error; TSD, total sleep disturbance.

The mediation analysis showed that sleep quality partially mediated the association between fish consumption and verbal IQ score, but it was not a mediator of the association between fish consumption and performance IQ score (Fig. 2). As shown in Fig. 2A, more frequent fish consumption was associated with elevated verbal IQ (step 1, total effect) when not considering the total sleep disturbance score in the multivariable model. After controlling for sleep disturbance, the magnitude of the fish consumption effect on verbal IQ was reduced and the corresponding P value became non-significant (step 4, direct effect), indicating that the effect of fish consumption on verbal IQ is partially mediated by overall sleep quality. Figure 2B shows that the adjustment for total sleep disturbance score did not affect the association between fish consumption and performance IQ, suggesting overall sleep quality was not a mediator explaining the relationship between fish consumption and performance IQ. Details on the 4-step mediation analysis are summarized in the Fig. 2.

## Discussion

Omega-3 fatty acids are essential dietary nutrients found in fish and have important implications for children’s health. In the present longitudinal study, children who consumed more dietary fish had both reduced sleep disturbances (better sleep quality) and better long-term cognitive outcomes. Such relationships still held significant after controlling for 13 sociodemographic covariates. Moreover, sleep was found to partially mediate the relationship between fish consumption and cognitive outcomes, suggesting that frequent fish consumption may improve sleep quality, which results in better long-term cognitive outcomes. These conclusions are supported by the finding of dose-response relationships between quantity of fish consumption and degree of increased IQ scores, relationships that were again found to be mediated by better sleep quality as indicated by less sleep disturbances. These findings thus have potentially significant implications for public health attempts to promote healthy dietary habits in children and adolescents.

Our findings regarding the relationships between fish consumption, sleep, and cognitive outcomes are consistent with the existing literature. First, our findings that fish consumption is significantly associated with improved cognitive function and performance in children adds to existing evidence from several European cohorts^7–9^. Second, the present study also confirms and makes important contributions to the existing knowledge on the relationship between fish consumption and sleep. While the effects of fish and omega-3 fatty acid intake on sleep have been shown in several studies in adults^29,30^ and infants^25,26^, less is known about this relationship in school-aged children. To our knowledge, Montgomery *et al*.^31^ is the only study that has examined this relationship in healthy children, demonstrating that in 395 children aged 7–9 years, higher serum DHA levels were associated with improved objective sleep measures including fewer wake episodes and more sleep each night^31^. Third, our findings also add to existing and well-established evidence that sleep is significantly associated with cognitive functioning^15–17,20^. As one example, sleep problems and fatigue have been associated with lower IQs^20^ whereas longer sleep duration has been associated with higher IQ and academic performance^16^.

Interestingly, we found that sleep mediated fish consumption and verbal IQ but not performance IQ. This partial mediation may reflect how the effects of fish consumption on sleep differentially affects specific neurocognitive domains rather than a global deficit. However, these potential effects on VIQ versus PIQ remain mixed and unclear. While Northstone *et al*.^44^ similarly found that fish consumption to be associated with VIQ but not PIQ, other studies have reported relationships with both VIQ and PIQ, suggesting that nutrients in fish enhance cognitive functioning in a global fashion^9^. Findings are similarly mixed regarding sleep’s effects on VIQ versus PIQ. For example, polysomnography studies have found associations between sleep spindles (which are believed to mediate cognitive functions) and PIQ, but not VIQ in children^16^. Still other studies in primary school children have found VIQ to be more vulnerable to the effects sleep deprivation and poor sleep quality^45^. It is thus possible, for instance, that fish consumption affects sleep quality or aspects of sleep that are more related to cognitive functions related to VIQ rather than PIQ. Clearly, more research identifying the relationships between cognitive functioning with both objective and subjective measures of sleep in children is warranted.

The robustness of the above findings is shown in several ways. First, fish consumption and sleep quality were assessed 1 to 3 years earlier than cognitive functions. Second, the three sets of relationships remained significant after controlling for 13 covariates. Lastly, the finding of dose-response relationships confirms and extends the findings based on comparisons of the three levels of fish consumption. Thus, we believe that the findings cannot be easily attributed to chance and that instead, they reflect a reliable relationship between early frequent fish consumption and later improved cognitive performance.

Importantly, our findings are also novel in demonstrating that sleep may serve as a mediator between frequent fish consumption and improved cognitive ability, providing an important mechanism by which fish consumption may affect cognitive functioning. To our knowledge, this is the first study to identify and demonstrate such a mediating effect. Omega-3 fatty acids are critical components of mammalian neural tissue and are known to have significant contributions to the growth and functioning of neural tissue^1^, including involvement in processes such as cortical glucose utilization^13^ and neural plasticity^14^. However, omega-3 fatty acids also appear to have direct effects on sleep, with animal models demonstrating the role of omega-3 fatty acids in critical sleep-regulating processes such as endogenous melatonin production^21–23^. Sleep, in turn, is hypothesized to affect cognitive function by facilitating learning, working memory, memory consolidation, and underlying neural plasticity in children^17^. Thus, it is reasonable to assume that fish consumption may improve neurodevelopmental outcomes not only by directly affecting cognitive processes, but also by improving sleep. Improved cognitive outcomes may also reflect the facilitation of processes occurring during sleep that are critical to cognitive performance.

Several potential limitations of the study should be recognized. First, although the current study is longitudinal, with early fish consumption/sleep measurement and later IQ testing, temporal ordering of the three constructs cannot be fully documented since fish consumption and sleep were measured at the same time. While mediation analysis tests a causal model, and while findings support the causal model proposed, we emphasize that our observational findings cannot document causality. A future prospective cross-lag longitudinal study measuring all three constructs at each time-point could provide a stronger test of the causal model that this study provisionally offers. Ideally, future randomized controlled trials which manipulate fish consumption and sleep will be launched to test the causal mechanism of the hypothesized model. In addition, because sleep outcomes were derived from subjective parental report, future research with both subjective and objective measures will be necessary to confirm our findings. Furthermore, we did not adjust for energy intake and use of omega-3 supplements since these measures were not assessed. Lastly, the specific types of fish consumed were not included in the analysis, due to limitations in children’s comprehension of fish types at this age. Future follow-up into adolescence in these children will include this component as they will then have a better understanding of fish varieties.

Our study found that fish consumption among school-aged children is associated with both improved sleep and cognitive ability, and that sleep partially mediated the relationship between fish consumption at age 9–11 years and cognitive ability as measured by IQ at age 12 years. These findings have important implications for public health efforts to promote healthy dietary habits in children and adolescents. More research is warranted to further explore the mechanisms through which intake of omega-3 fatty acids may contribute to improved neurodevelopment and cognitive function.
